# Good neurological outcome despite very low regional cerebral oxygen saturation during resuscitation—a prospective preclinical trial in 29 patients

**DOI:** 10.1186/s13049-016-0234-3

**Published:** 2016-04-06

**Authors:** Christian Storm, Alexander Wutzler, Lars Trenkmann, Alexander Krannich, Sabrina von Rheinbarben, Fridolin Luckenbach, Jens Nee, Natalie Otto, Tim Schroeder, Christoph Leithner

**Affiliations:** Department of Internal Medicine, Nephrology and Intensive Care, Charité-Universitätsmedizin Berlin, Augustenburgerplatz 1, 13353 Berlin, Germany; Department of Cardiology, Charité-Universitätsmedizin Berlin, Augustenburgerplatz 1, 13353 Berlin, Germany; Department of Biostatistics, Berlin Institute of Health, Clinical Research Unit, Charité-Universitätsmedizin Berlin, Augustenburgerplatz 1, 13353 Berlin, Germany; Department of Neurology, Charité-Universitätsmedizin Berlin, Augustenburgerplatz 1, 13353 Berlin, Germany

**Keywords:** Cardiac arrest, Outcome, Near infrared spectroscopy, Out-of-hospital cardiac arrest

## Abstract

**Background:**

Noninvasive regional cerebral oxygen saturation (rSO_2_) measurement using near-infrared spectroscopy (NIRS) might inform on extent and duration of cerebral hypoxia during cardiopulmonary resuscitation (CPR). This information may be used to guide resuscitation efforts and may carry relevant early prognostic information.

**Methods:**

We prospectively investigated non-traumatic out-of-hospital cardiac arrest (OHCA) patients on scene. NIRS was started either during CPR or shortly after (<2 min) return of spontaneous circulation (ROSC) by emergency medical service (EMS). Outcome was determined at intensive care unit (ICU) discharge and 6 months after cardiac arrest.

**Results:**

A total of 29 OHCA patients were included. In 23 patients NIRS was started during CPR and in 6 patients immediately after ROSC. 18 (62.1 %) patients did not reach ROSC. Initial rSO_2_ during CPR was very low (<50 % in all 23 patients, < 30 % in 19 of 23 patients) with no significant difference between patients achieving ROSC and those who did not. Of five patients with ROSC, in whom NIRS was recorded during CPR, two reached a good six-months outcome (initial rSO_2_ 22 %) and three died during the ICU stay (initial rSO_2_ 15, 16 and 46 %). In six patients with NIRS started immediately after ROSC (<2 min), rSO2 was substantially higher (54–85 %) than in patients during CPR (*p* = 0.006).

**Discussion and conclusion:**

Initial frontal brain rSO_2_ determined by NIRS during CPR was generally very low and recovered rapidly after ROSC. Very low initial rSO_2_ during CPR was compatible with good neurological outcome in our limited cohort of patients. Further studies are needed to assess in larger cohorts and more detail the implications of very low initial rSO_2_ during CPR on scene.

## Background

The extent of hypoxic encephalopathy (HE) largely determines outcome in patients after cardiac arrest and resuscitation. In principle, the severity and duration of brain hypoxia during cardiac arrest (CA) can be determined by noninvasive near-infrared spectroscopy (NIRS) during resuscitation. The brain extracts only around one third of the oxygen delivered under physiological conditions, thus normal values of frontal brain rSO_2_ determined by NIRS monitors are around 60–80 %. As the brain has no oxygen reserves and demand is high, brain oxygen saturation drops to very low values very shortly after cardiac arrest [1]. During total oxygen depletion, nonoxidative metabolism can deliver sufficient ATP to prevent irreversible neuronal damage for a few minutes. Hypoxic encephalopathy can only be prevented by restoring oxygen delivery via effective resuscitation before irreversible damage has occured. NIRS may be used to guide resuscitation efforts by indicating the achieved cerebral oxygenation which is one of the primary targets of CPR [1, 2]. Few studies have measured regional brain oxygen saturation using NIRS in patients during resuscitation [3]. The study settings were heterogeneous. Most importantly, large studies have been reported on NIRS started upon hospital arrival as opposed to studies which measured NIRS during CPR on scene [4–8]. Likely, measurements started during CPR upon hospital arrvial cover a much later time point after cardiac arrest as compared to measurements started during CPR on scene and thus, results obtained in these two different settings need to be compared with great care. In principle, a lower threshold for the initial rSO_2_ during resusciation may exist below which ROSC or survival with good outcome are rare [8]. On the other hand, brain rSO_2_ may change rapidly during CPR and full recovery of brain tissue is possible if the periods of severe hypoxia are short enough to allow for survival of neurons via non-oxidative metabolism [9]. To contribute to the understanding of prognostic implications of brain oxygen saturation during CPR, we performed a prospective study on out-of-hospital cardiac arrest patients using near-infrared spectroscopy to determine frontal brain rSO_2_ during CPR in the field at the earliest possible time point.

## Methods

The local ethics committee of the Charité-Universitätsmedizin Berlin approved the study protocol and the trial was registered (www.clinicaltrials.gov: NCT 01531426). For all survivors a healthcare proxy was contacted to give written informed consent as all cardiac arrest survivors were unconscious on admission. Nontraumatic cardiac arrest patients of cardiac and non-cardiac etiology were enrolled between January 2012 and January 2013.

### Pre-hospital treatment

Advanced cardiac life support (ACLS) was performed according to current guidelines.

NIRS monitoring started out-of-hospital and was continued until the end of the rewarming procedure (approximately 41 h in total) in all survivors. The INVOS monitor (INVOS 5100 C; Covidien; Mansfield, USA) was used. Of note, the lower rSO_2_ detection limit of the monitor was 15 % in our study.

The two surface sensors were placed on the forehead for detection of bilateral frontal cerebral oxygen saturation. The monitor detects the absorption of light at wavelengths of 724 nm and 810 nm and calculates regional hemoglobin oxygen saturation (rSO_2_). For analysis of the recorded spectroscopy data the software package provided by COVIDIEN was used (INVOS Analytics Tool, Version 1.2). The mean value between right and left sensor was calculated. For the purposes of our preclinical study, we determined the first reliably measured value and refer to it as ‘initial rSO_2_’. We have previously reported rSO_2_ measurements continued until the end of hypothermia treatment [10]. An additional paramedic not involved in patient care performed the trial-related monitoring, carefully avoiding any interference with the ACLS team. The NIRS sensors were placed during cardiopulmonary resuscitation or within two minutes after ROSC. Forehead and upper part of the body positioning was standardized at 30° in all patients during the whole period of treatment and this position was adopted immediately after ROSC at the scene.

### Hospital treatment

All patients received cardiac arrest treatment according to our written local standard operating procedure and post-resuscitation care according to current guidelines as described in detail elswhere (24 h at 33 °C followed by slow rewarming 0.25°/h) [10].

Hypothermia was performed with a computer controlled feedback surface-cooling device (Arctic Sun; C.R.BARD).

### Outcome assessment

For survivors, outcome was assessed at discharge from the ICU and at 6-months follow up by the Pittsburgh Cerebral Performance Category (CPC) Scale. Because both short and long-term outcomes were assessed, we defined good outcome as CPC 1–2 and poor outcome as CPC 3–5. For the 6-months follow up, the patient or a proxy was contacted by telephone to assess CPC. Data on mortality was obtained by the German residents registry.

### Statistics

Statistical analysis was performed using SPSS (IBM SPSS Version 20) and R (R 3.1.2, The R Project). Due to the low number of patients, data are presented as individual values and as median and quartiles (IQR) or absolute numbers and percent. For comparison of rSO_2_ between different groups a Wilcoxon-Mann–Whitney-Test was performed. A significance level of α = 0.05 was used.

## Results

**Fig. 1:**
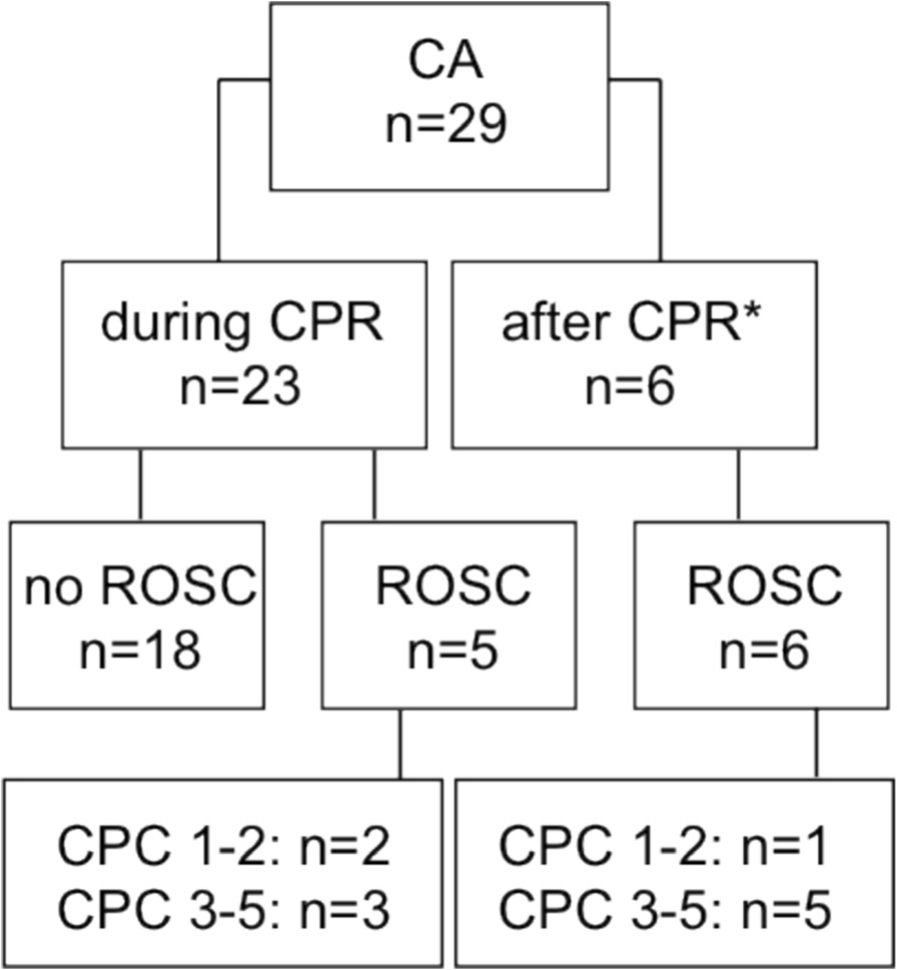
Flow chart of the trial population. CPR cardiopulmonary resuscitation; ROSC return of spontaneous circulation; *NIRS starteted after ROSC within ≤ 2 min; CPC cerebral performance category (1–2 good neurological outcome; CPC 3–5 poor neurological outcome); CA cardiac arrest

In 29 patients data recorded by the EMS during CPR (*n* = 23) or shortly after ROSC (*n* = 6) were available (study flow chart, Fig. 1). Baseline characteristics of all patients are given in Table 1. 18 patients (62 %) did not achieve ROSC. 11 patients (38 %) were admitted to the ICU after ROSC. Of those, three had good outcome (CPC 1–2) at ICU discharge and eight had poor outocme (one CPC 3, seven CPC 5) (Table 2). All patients discharged (*n* = 4) were followed up at 6 months. The three patients discharged with CPC 1–2 continued with a good neurological status (all CPC 1), one patient was discharged with CPC 3 and had died at 6 month follow up.

**Table 1:** Baseline characteristics

	Post ROSC	Pre ROSC	No ROSC
*n*	6	5	18
Age (years) (mean (sd))	68 (9)	66 (7)	61(14)
Sex male *n* (%)	6 (100)	4 (80)	14 (78)
APACHE score (median [IQR])	28 [20, 36]	37 [20, 40]	-
Shockable rhythm n (%)			
Ventricular fibrillation	2 (33)	1 (20)	4 (22)
Asystolic	1 (17)	3 (60)	9 (50)
EMD	3 (50)	1 (20)	5 (27)
Time to ROSC (min) (median [IQR])	19 [11, 27]	12 [9, 16]	-
Total epinephrine dose (mg) (median [IQR])	2 [0.5, 4.25]	1 [1, 2]	7 [4, 9.5]
Haemoglobin (g/dl) (median [IQR])	12 [11, 14]	13 [12, 15]	-
Lactate (median [IQR])	29 [27, 45]	52 [43, 60]	-
Time on ventilator (hours) (median [IQR])	382 [164, 458]	212 [73, 388]	-
Length of ICU stay (days) (median [IQR])	17 [8, 20]	14 [3, 16]	-

Data are given as median and interquartile range (IQR) or absolute numbers and percent. ‘Post ROSC’ designates the group of patients in which NIRS was started shortly after ROSC, ‘pre ROSC’ designates the group with NIRS started during CPR and who achieved ROSC, ‘no ROSC’ the group with NIRS during CPR but no ROSC. APACHE Acute Physiology And Chronic Health Evaluation; OHCA out-of-hospital cardiac arrest; ROSC return of spontaneous circulation; ICU intensive care unit; EMD electromechanical dissociation; PaO_2_ arterial oxygen partial pressure; SO_2_ peripheral oxygen saturation; PaCO_2_ arterial carbon dioxide partial pressure; FiO_2_ fraction of inspired oxygen

**Table 2:** Data of *n* = 29 patients included by EMS divided into NIRS during CPR without ROSC (*n* = 18), ROSC (*n* = 5) and NIRS started after ROSC (*n* = 6)

	rSO_2_ EMS data *n* = 29						
	rSO_2_ (%)	Outcome		tROSC (min)	First rhythm	Age (years)	Sex
rSO_2_ during CPR *n* = 23		Discharge	6 month				
*Pre ROSC n* = *5*							
1	46 %	CPC 5		16	asystole	59	male
2	22 %	CPC 1	CPC 1	8	asystole	73	male
3	15 %	CPC 5		12	asystole	65	female
4	16 %	CPC 5		19	PEA	72	male
5	22 %	CPC 1	CPC 1	9	VF	58	male
No ROSC *n* = 18							
1	15 %	CPC 5			asystole	50	female
2	15 %	CPC 5			asystole	72	female
3	15 %	CPC 5			asystole	89	female
4	15 %	CPC 5			PEA	52	male
5	15 %	CPC 5			VF	58	male
6	15 %	CPC 5			VF	73	male
7	15 %	CPC 5			VF	55	male
8	41 %	CPC 5			PEA	64	male
9	16 %	CPC 5			asystole	46	male
10	20 %	CPC 5			PEA	63	female
11	22 %	CPC 5			asystole	65	male
12	15 %	CPC 5			VF	75	male
13	35 %	CPC 5			asystole	78	male
14	15 %	CPC 5			asystole	43	male
15	42 %	CPC 5			asystole	61	male
16	15 %	CPC 5			asystole	47	male
17	15 %	CPC 5			PEA	76	male
18	15 %	CPC 5			PEA	42	male
Post ROSC *n* = 6							
1	63 %	CPC 5		19	asystole	73	male
2	71 %	CPC 5		30	VF	61	male
3	57 %	CPC 5		60	PEA	68	male
4	54 %	CPC 5		5	PEA	84	male
5	80 %	CPC 1	CPC 1	9	VF	61	male
6	85 %	CPC 3	CPC 5	18	PEA	60	male

Initial rSO_2_, outcome, time to ROSC, first rhythm, age and sex are given

### Initial regional oxygen saturation and ROSC

**Fig. 2:**
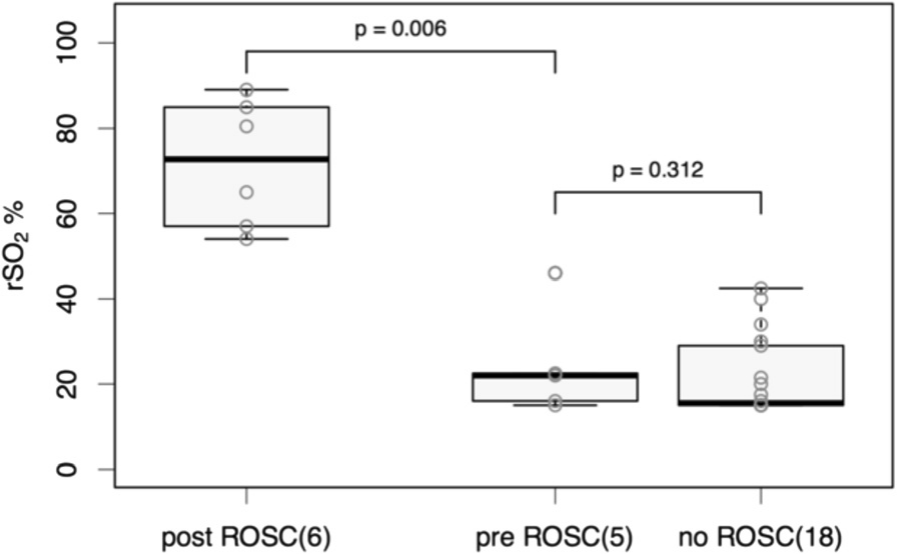
Initial rSO_2_ (median and IQR) for patients in whom NIRS was started within two minutes after ROSC (‘post-ROSC’), patients in whom NIRS was started during CPR and who achieved ROSC (‘pre-ROSC’) and in patients during CPR who did not achieve ROSC (‘no ROSC’)

Of 23 patients with NIRS started during CPR,18 never reached ROSC. Clinical details and initial rSO_2_ of the individual patients are shown in Table 2. Figure 2 illustrates initial rSO_2_ of patients who did not achieve ROSC (‘no ROSC’), those in whom NIRS was started during CPR and who achieved ROSC (‘pre-ROSC’) and those in whom NIRS was started within two minutes after ROSC (‘post ROSC’). Initial rSO_2_ in the patients with no ROSC was generally very low (median 16 %, IQR 15–29 %). Initial rSO_2_ was also low in the five patients with ROSC in whom NIRS was started during CPR (15, 16, 22, 23 and 46 %, Table 2). The difference was not statistically significant (*p* = 0.312). In six patients NIRS monitoring could not be started pre-ROSC but immediately after ROSC. Initial rSO_2_ was substantially higher (54–85 %) in these patients as compared with patients with pre-ROSC NIRS monitoring (*p* = 0.006, Fig. 2).

### Initial regional oxygen saturation and neurological outcome

Five patients with NIRS recording during CPR reached ROSC. Two of those patients had a good outcome (CPC 1, both at discharge and at six months). The initial rSO_2_ of both of these two patients was 22 %. Three patients with ROSC in whom NIRS was recorded during CPR died during ICU stay. The initial rSO_2_ of these patients was 15, 16 and 46 %; Table 2). In six patients monitoring started approximately two minutes after ROSC. One patient had a good outcome (CPC1) and of the remaining five patients, four died during the ICU stay and one had CPC3 at ICU discharge and had died at six months follow-up. The patient with good outcome had a high initial post-ROSC rSO_2_ (80 %).

### Withdrawal of treatment

In 5 patients ICU treatment was withdrawn due to severe hypoxic encephalopathy (*n* = 4) and multi-organ failure (*n* = 1). Withdrawal of treatment for hypoxic encephalopathy was done after repetitive neurological examination, neurophysiologcial, radiological and laboratory testing if available (including somatosensory evoked potentials (SSEP), neuro-specific enoloase (NSE), brain computed tomography (CT) and electroencephalography EEG) following a standardized pathway. Brain oxygen saturation measurements were not used for prognostic purposes.

## Discussion

Our main findings are: (1) Patients with initially very low regional cerebral oxygen saturation (as low as 22 % determined by a commercially available near-infrared spectroscopy monitor) during out-of-hospital cardiac arrest and resuscitation may survive with good outcome. (2) Intial rSO_2_ during CPR obtained immediately after arrival of EMS was generally low and did not allow to predict ROSC or neurological outcome. (3) Immediately (<2 min) after ROSC, rSO_2_ was substantially higher at or close to physiological values (54-85 % in six patients).

### Pre-ROSC rSO_2_

The very low regional frontal brain oxygen saturation observed in our study is in line with previous reports. For example, in an early report with NIRS during CPR, Newman and coworkers found no detectable cerebral oxygen saturation during CPR in all of sixteen subjects [11]. Parnia and coworkers also found very low initial rSO_2_ (15–21 %) determined by NIRS during CPR in 15 cardiac arrest patients. Increasing rSO_2_ with ongoing CPR was observed which was associated with ROSC in their study [12]. In another trial, Parnia and coworkers demonstrated low rSO_2_ during manual chest compression (median rSO_2_ 24 %) and significantly higher values in patients treated with an automated chest compression device (median rSO_2_ 53 %) [13]. Schewe and coworkers reported similar results [4]. Kämäräinen also demonstrated very low rSO_2_ during manual CPR which did not substantially increase by improving manual CPR technique [14]. In line with our results, Genbrugge et al. reported no significant difference between initial rSO_2_ in patients achieving ROSC compared to those who did not when NIRS was started during CPR on scene [5]. Taken together, these studies consistently show a very low cerebral oxygen saturation as measured by commercially available NIRS monitors during cardiac arrest and CPR. Clearly, brain hypoxia during CPR causes severe hypoxic encephalopathy in many patients. However, when interpreting rSO_2_ measurements during CPR it needs to be kept in mind that compared to the cerebral metabolic rate of oxygen in an awake state the brain needs much less oxygen to simply maintain cell viability. Furthermore, regional brain rSO_2_ does not directly inform on the amount of oxygen delivered to the brain and thus low rSO_2_ obtained at a single time point cannot predict the extent of hypoxic encephalopathy. It may be, however, that integrating information on duration and extent of hypoxia measured by NIRS may allow for a largely reliable prediction of outcome and may be used in the future among other parameters in the decision to continue or stop resucitation efforts.

### Post ROSC rSO_2_

In our study, the six patients with rSO_2_ measurement started immediately after ROSC (<2 min) showed regional frontal brain oxygenation at or close to physiological values (54–85 %). The rapid increase of rSO_2_ following ROSC suggested by this finding is in line with previous reports. E.g., Genbrugge and coworkers demonstrated rSO_2_ changes from 25–30 % immediately pre-ROSC up to 60–70 % two minutes post-ROSC in a cardiac arrest patient [9]. Kämäräinen et al. found a median rSO_2_ of 60 % in seven patients eight minutes after ROSC [14]. Ito and coworkers found substantially higher rSO_2_ in cardiac arrest patients with detectable pulses (ROSC) upon hospital arrival (51 %) as compared to those without (19 %). Collectively, these studies indicate that rSO_2_ rapidly increases after ROSC to median/mean values close to physiological rSO_2_.

### Initial rSO_2_ and outcome prediction

In a recent large multicenter trial, Ito and coworkers found very low initial rSO_2_ (15 % as determined by a commercially available NIRS monitor, representing the lower measurement threshold) in three patients with good neurological outcome [6]. Only 29 out of 672 patients in this study had a good outcome. In contrast to our study with NIRS started on scene, Ito and coworkers started rSO_2_ measurements upon hospital arrival after EMS transfer with ongoing CPR. Termination of CPR is not allowed for EMS in Japan. Our cohort was collected in a large urban area with a short response time of EMS. Thus, it is very likely that in our study NIRS was started much earlier in the course of CPR than in the study by Ito and coworkers. A subset of patients in the study by Ito and coworkers had already achieved ROSC when NIRS was started upon hospital arrival which explains the more frequent finding of high initial rSO_2_ in their study [6]. Taken together, our study and the study by Ito and coworkers underscore that patients may recover with good neurological outcome despite very low frontal brain rSO_2_ recorded during CPR on scene as well as upon hospital arrival. Very recently, Nishiyama and coworkers reported a very large study on NIRS during CPR at hospital arrival. Their results indicate that the proportion of patients with good neurological outcome at the lowest measured initial rSO_2_ (15 %, the lower detection limit of the monitor) is very low. Although our patient cohort is smaller, our results indicate that initial rSO_2_ obtained during CPR at hospital arrival and initial rSO_2_ obtained during CPR on scene (in a setting with short EMS response time) bear different prognostic implications. Before NIRS started during CPR on scene may be used for prediction of outcome and in decisions to terminate CPR, further large studies on the relationship between duration and extent of regional brain hypoxia as measured by NIRS and outcome are needed.

## Limitations

Because ROSC is achieved in less than half of patients with cardiac arrest and less than half of those have had a good neurological outcome, the number of patients with good neurological outcome included was small. Thus, we could not determine the distribution of initial rSO_2_ during CPR in a large cohort of patients with good neurological outcome. Larger studies are desirable to determine whether our two cases of good neurological outcome despite very low initial rSO_2_ are exceptional. Furthermore, we did not record the course of rSO_2_ during resuscitation until ROSC which would have allowed for am more detailed investigation. It is also possible that the cause of arrest may influence the relationsship between rSO_2_ recorded during CPR and outcome. E.g., patients with respiratory arrest may have significant hypoxia already prior to the cardiac arrest which could influence the periode between cardiac arrest and irreversible neuronal damage. We did not record the time between collapse/emergency call and first NIRS recording with sufficient precision. This may influence the relationsship between initial rSO_2_ and outcome or chance of ROSC and it would be interesting to investigate this question in future studies. Calculation of regional brain oxygen saturation using noninvasive near-infrared spectroscopy is complex and results may differ between different commercially available NIRS-monitors due to different rSO_2_ calculation algorithms. The NIRS signal does not originate exclusively from brain tissue and therefore, confounding by extracerebral sources is possible. The sample volume within the brain contains arteries, capillaries and veins with different oxygen tensions. The composition of the sample volume varies from patient to patient. Thus, the absolute values for rSO_2_ reported in this study need to be interpreted with caution and do not represent average brain tissue oxygenation.

## Conclusion

Initial brain oxygen saturation is genearlly very low in cardiac arrest patients immediately after arrival of EMS. Very low initial brain oxygen saturation is compatible with ROSC and with good neurological outcome and should not be regarded as an absolute poor prognostic sign.

## References

[1] Genbrugge C, Boer W, Meex I, Jans F, Dens J, De Deyne C (2014). Cerebral tissue saturation, the next step in cardiopulmonary resuscitation management?. Crit Care..

[2] Parnia S (2012). Cerebral oximetry - the holy grail of non-invasive cerebral perfusion monitoring in cardiac arrest or just a false dawn?. Resuscitation..

[3] Sanfilippo F, Serena G, Corredor C, Benedetto U, Maybauer MO, Al-Subaie N (2015). Cerebral oximetry and return of spontaneous circulation after cardiac arrest: A systematic review and meta-analysis. Resuscitation..

[4] Schewe JC, Thudium MO, Kappler J, Steinhagen F, Eichhorn L, Erdfelder F (2014). Monitoring of cerebral oxygen saturation during resuscitation in out-of-hospital cardiac arrest: a feasibility study in a physician staffed emergency medical system. Scandinavian journal of trauma, resuscitation and emergency medicine.

[5] Genbrugge C, Meex I, Boer W, Jans F, Heylen R, Ferdinande B (2015). Increase in cerebral oxygenation during advanced life support in out-of-hospital patients is associated with return of spontaneous circulation. Crit Care..

[6] Ito N, Nishiyama K, Callaway CW, Orita T, Hayashida K, Arimoto H (2014). Noninvasive regional cerebral oxygen saturation for neurological prognostication of patients with out-of-hospital cardiac arrest: a prospective multicenter observational study. Resuscitation..

[7] Fukuda T, Ohashi N, Nishida M, Gunshin M, Doi K, Matsubara T (2014). Application of cerebral oxygen saturation to prediction of the futility of resuscitation for out-of-hospital cardiopulmonary arrest patients: a single-center, prospective, observational study: can cerebral regional oxygen saturation predict the futility of CPR?. The American journal of emergency medicine..

[8] Nishiyama K, Ito N, Orita T, Hayashida K, Arimoto H, Beppu S (2015). Regional cerebral oxygen saturation monitoring for predicting interventional outcomes in patients following out-of-hospital cardiac arrest of presumed cardiac cause: A prospective, observational, multicentre study. Resuscitation..

[9] Genbrugge C, Dens J, Meex I, Boer W, Jans F, De Deyne C (2013). Cerebral saturation monitoring during cardiopulmonary resuscitation should be used as dynamic, rather than static, information. Resuscitation..

[10] Storm C, Leithner C, Krannich A, Wutzler A, Ploner CJ, Trenkmann L (2014). Regional cerebral oxygen saturation after cardiac arrest in 60 patients-A prospective outcome study. Resuscitation..

[11] Newman DH, Callaway CW, Greenwald IB, Freed J (2004). Cerebral oximetry in out-of-hospital cardiac arrest: standard CPR rarely provides detectable hemoglobin-oxygen saturation to the frontal cortex. Resuscitation..

[12] Parnia S, Nasir A, Shah C, Patel R, Mani A, Richman P (2012). A feasibility study evaluating the role of cerebral oximetry in predicting return of spontaneous circulation in cardiac arrest. Resuscitation..

[13] Parnia S, Nasir A, Ahn A, Malik H, Yang J, Zhu J (2014). A feasibility study of cerebral oximetry during in-hospital mechanical and manual cardiopulmonary resuscitation*. Crit Care Med..

[14] Kamarainen A, Sainio M, Olkkola KT, Huhtala H, Tenhunen J, Hoppu S (2012). Quality controlled manual chest compressions and cerebral oxygenation during in-hospital cardiac arrest. Resuscitation..

